# Expression of circadian clock gene human Period2 (hPer2) in human colorectal carcinoma

**DOI:** 10.1186/1477-7819-9-166

**Published:** 2011-12-13

**Authors:** Yaping Wang, Luchun Hua, Chao Lu, Zongyou Chen

**Affiliations:** 1 Department of Surgery, Huashan Hospital, Fudan University, Shanghai, China; 2 Department of Physiology and Pathophysiology, Shanghai Medical College, Fudan University, Shanghai, China

**Keywords:** circadian, clock gene, human Period2 (hPer2), colorectal carcinoma, expression

## Abstract

**Background:**

Recent studies have shown that disruption of circadian rhythms is one of the tumor promoting factors which contribute to mammalian cancer development and progression, but very little is known about the molecular changes of circadian genes in colorectal carcinoma (CRC). Thus, in this study, changes in the expression of human Period2 (hPer2), one of the key circadian clock regulators, in CRC and its correlation with prognosis were investigated.

**Methods:**

Immunohistochemical (IHC) staining and real-time PCR for hPer2 were performed for 38 CRC cases.

**Results:**

IHC analysis detected positive staining for hPer2 in 81.6% (31/38) of CRC tissues and 97.4% (37/38) of surrounding non-cancerous tissues (P < 0.05). Most colorectal cells in non-cancerous tissues were homogeneously stained. In contrast, in the paired cancerous tissues, a heterogeneous pattern was found with a significant portion of cancer cells displaying negative or weak hPer2 staining. In over 60% cases (24/38), the staining for hPer2 was much stronger in non-cancerous cells than in the paired cancerous cells. Well-differentiated cancer cells are more likely to maintain hPer2 expression than poorly-differentiated ones. Furthermore, associations of decreased hPer2 levels with patients' age, histological grade, TNM stage and expression of nucleus proliferation related antigen: Ki67 were also detected (P < 0.05). Expression of hPer2 did not correlate with that of either p53 or C-erB-2. Similar to hPer2 protein expression, quantitative RT-PCR for hPer2 also showed decreased mRNA expression in CRC.

**Conclusion:**

These results suggest a role for hPer2 in normal colorectal cell function and the potential deregulation of hPer2 expression in the development, invasion, and metastasis of CRC.

## Background

Various kinds of living organisms exhibit behavioral and physiological circadian rhythms, allowing them to adapt to the daily cycle of light and dark [1]. At the molecular level, the rhythms of the circadian clock are controlled by the interaction between positive and negative feedback loops consisting of several key clock regulators [2,3]. A model encompassing a feedback system involving heterodimer transcriptional factors (Clock and Bmal1), two cryptochromes (Cry1 and Cry2), and three Period (Per1, Per2, and Per3) regulators has been widely described.

Among all the known clock genes, Per2 has been shown to play an important role in tumor progression [4,5]. Dysregulation of hPer2 gene has been found in many types of human cancers [6,7]. Genetic studies also showed that mice with dysfunctional circadian rhythms are prone to many kinds of cancer developing [8,9]. Mice deficient in mPer2 showed significant higher tumor incidence [10]. Moreover, functional studies found that overexpression of Per2 inhibited cancer cell growth in both culture system and xenograft mouse model [11-13]. In terms of the mechanisms, C-erB-2 and p53 were suggested to act as the downstream players for hPer2 in the course of tumor progression [7,10,14]. Both the C-erbB-2 oncogene and the p53 tumor-suppressor gene integrate numerous signals that control cell proliferation and survival. As when a highly connected node in the internet breaks down, the activation of C-erbB-2 or disruption of p53 leads to severe consequence of tumorigenesis [15-17]. Although hPer2 is implicated as a tumor suppressor, the expression pattern of hPer2 in cancer is not quite clear. Whether hPer2 expression is associated with other tumor-associate proteins such as C-erB-2 and p53 in human CRC remains uncertain.

Colorectal cancer is one of the most commonly seen malignancies and the leading cause of cancer related death worldwide. About 141,210 new cases and 49,380 deaths were expected for 2011 in the United States [18]. In China, CRC is the fourth leading cause of cancer mortality in big cities and the fifth in countryside. However, in Shanghai, CRC incidence and mortality rates ranked the second and third respectively for female [19]. Since surgical approaches and conventional therapeutic have not been able to fully control the outcomes of CRC, there is an urgent need to develop more effective treatments.

The circadian rhythm is interconnected with many aspects of cellular functions such as cell proliferation, migration and differentiation, thus, it plays a major role in regulating the digestive system [20,21]. Many laboratories have reported strong evidence about the beneficial effects of chronotherapy, which refer to chemotherapy delivery according to the circadian rhythm [22,23]. Phase I-III clinical trials have shown that chronotherapy significantly increased tolerance to high doses of chemotherapy drugs and improved clinical response in patients with metastatic colorectal cancer [24,25]. These findings further interest us to explore the relationship between circadian rhythms and CRC at molecular level. In the present study, we used immunohistochemical staining and real-time PCR to characterize the role of hPer2 in the development of human CRC.

## Methods

### Tissue samples

38 resected CRC samples and paired non-cancerous tissues were obtained from the patients of Huashan hospital, Shanghai, China, undergone radical surgery for colorectal cancer (20 males and 18 females). All patients had a definitive pathological diagnosis and accepted neither chemotherapy nor radiotherapy before operation. The samples were surgically obtained at the following points: 23 cases between 10:00 and 12:00, 8 cases between 12:00 and 14:00, 3 cases between 14:00 and 16:00, 3 cases between 16:00 and 18:00, and 1 case at 22:00. After surgical removal, the samples were preserved in liquid nitrogen immediately and then moved to the - 80°C freezer for long term storage. The age of the patients, tumor site, tumor type, histological grade and TNM stage according to AJCC classification were recorded.

### Immunohistochemistry

Frozen tissues were cut into 4 μm- thick sections and adhered to slides at - 20°C, fixed by acetone and stored in the - 20°C refrigerator. Endogenous peroxidase activity was blocked by incubation of sections in 3% H_2_O_2_/methanol. Sections were covered with pre-block solution at 37°C for 30 minutes, followed by Per2 antibody (1:50, Santa Cruz biotechnology) incubation for 60 minutes at 37°C. After a brief wash with PBS, biotinylated secondary antibody was added with 60 minutes at 37°C. After several washes with PBS, staining was achieved using 3, 3'-diaminobenzidine for about 5~10 minutes. Finally, slides were counterstained with Mayer's hemalum and mounted. Appropriate positive and negative controls were also included. The frozen sections were also stained with C-erB-2(sp3, dako), p53 (DO-7, dako) and Ki67 (Ki-S5, dako) by the same method.

### Grading of immunohistochemical findings

Immunohistochemical findings were scored depending on the extent and intensity of staining. All sections were graded by two independent investigators without knowing the patient's outcome. At least 10 randomly selected high - power fields were scored, and the average score was recorded.

### RNA extraction and first-strand cDNA synthesis

Preparation of total RNA from tissue samples was done using the Trizol reagent (Invitrogen Co.). The amount of total RNA was determined by UV spectrophotometry, and RNA integrity was assessed by agarose gel electrophoresis. First-strand cDNA was prepared with oligo-dT primers using a commercial cDNA synthesis kit (Fermentas RevertAId™). The cDNA was then amplified for 33 cycles with specific primers for Per2 and GAPDH which was utilized as an internal reference (table 1). Sequence data were analyzed using the Basic Local Alignment Search Tool (BLAST) software located at the National Centre for Biotechnology Information (NCBI) web site: http://www.ncbi.nlm.nih.gov web site.

**Table 1:** Primer pairs used for real-time PCR

	primer (5'-3')	Product length
hPer2	Forward:TCCAGTGGACATGAGACCAA Reverse:CGCTACTGCAGCCACTTGTA	186 bp
GAPDH	Forward:AACCTGCCAAATATGATGAC Reverse:ATACCAGGAAATGAGCTTGA	191 bp

The PCR reactions containing SYBR-green were amplified on a Corbett Real Time PCR machine (Bio-Rad, USA). After reverse transcription, the cDNA was diluted with sterile deionized water to 10-fold. The reaction mixtures for PCR contained 5 mL of diluted cDNA, 1 mL of 10 mmol/L primers (0.5 mL of forward primers, 0.5 mL of reverse primers), 12.5 mL of real-time PCR Master Mix (TOYOBO Co.), and 6.5 mL of H_2_O, into a final volume of 25 mL. After an initial incubation for 5 min at 94°C, the samples were subjected to 33 cycles of amplification (denaturation at 94°C for 30 sec, primer annealing at 52°C for 45 sec, extension at 72°C for 45 sec) with the final incubation for 15 min at 72°C.

After each real-time PCR, melting profiles as well as agarose gel electrophoresis of each sample were done to rule out the possibilities of nonspecific PCR products and primer dimers. Each 4 μL of PCR product was electrophoresed on 2% agarose gel and visualized with UV transilluminator. Data was acquired as threshold cycle (ΔCt) value. As internal standard to normalize mRNA levels for differences in sample concentration and loading, amplification of GAPDH was used. Standard curves were constructed for each target gene and internal control by plotting ΔCt values versus log cDNA dilution. Because the amplification efficiencies of target genes and internal control were equal, the relative changes of target gene expression in tumor cells compared with normal colorectal mucosa (ΔCt calibrator value) were calculated using the equation 2^-ΔΔCt^, where ΔΔCt = ΔCt (cancer)-Δ(non-cancer). The ΔCt values were determined by subtracting the average GAPDH Ct value from the average target gene Ct value.

### Statistical analysis

Comparison between the expression of hPer2 mRNA levels in normal colorectal mucosa and tumor tissue of patients was done by paired t-test, Ki67 level with hPer2 protein expression was done by t-test, and χ^2^-test or Fisher exact test was performed to analyze the correlations of hPer2 protein levels with clinical and pathologic parameters. SPSS, 16.0 was used in the above data analysis. P-values of less than 0.05 were considered to be statistically significant.

## Results

### Immunohistochemical analyses of hPer2 protein expression

**Figure 1:**
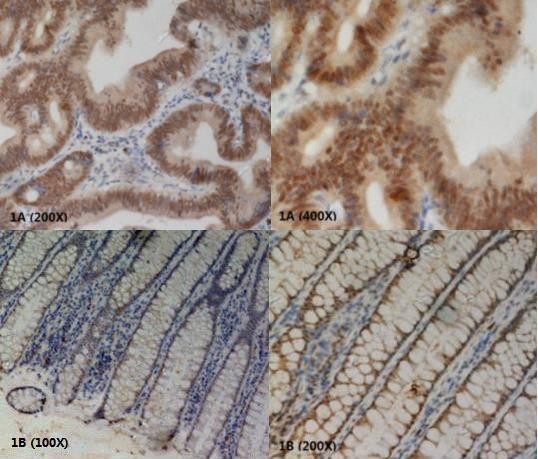
**1A Immunohistochemical analyses of hPer2 protein positively expression in the colorectal cancerous tissues(200×/400×)**. 1B Immunohistochemical analyses of hPer2 protein positively expression in the paired non-cancerous colorectal tissues(100×/200×).

**Figure 2:**
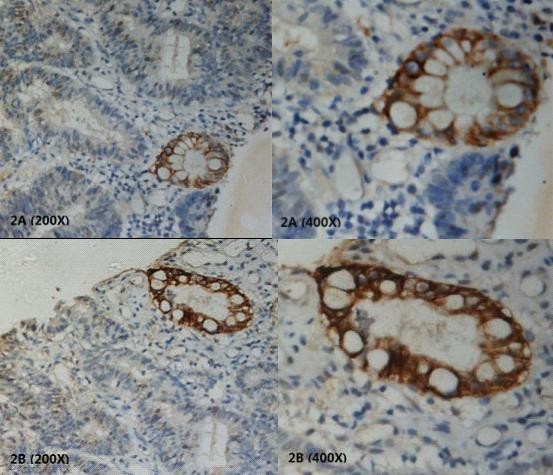
**Immunohistochemical analyses of hPer2 protein heterogeneous expression in the colorectal cancerous tissues, positively stained in well-differentiated cells**. 2A and 2B are two different individuals in which this interesting phenomenon can be found(200×/400×).

To investigate whether hPer2 gene was deregulated in colorectal cancer, we first examined hPer2 protein expression in 38 paired colorectal cancerous and non-cancerous tissues by IHC staining. hPer2 expression was found in both cytoplasm, and nucleus. The results revealed that positively stained cells can be found in 81.6% (31/38) of colorectal carcinoma tissues (Figure 1A) and 97.4% (37/38) of surrounding non-cancerous tissues (Figure 1B) (P < 0.05). Most colorectal cells in non-cancerous tissues were homogeneously stained. In contrast, in the paired cancerous tissues, there was a significant portion of cancer cells displaying negative or weak staining. This heterogeneous staining phenomenon was frequently observed in our study. Moreover, well-differentiated cancer cells tend to have comparable hPer2 level with that in non-cancerous cells (Figure 2), suggesting that loss of hPer2 expression may associate with increased aggressiveness. For these 38 cases, the hPer2 protein expression in colorectal tissues exhibited three different patterns: typeI (24 cases): the staining for hPer2 was much stronger in non-cancerous cells than in the paired cancerous cells; type II (12 cases): there were no significant differences between the staining of cancerous and non-cancerous cells; type III (2 cases): the staining for hPer2 was stronger in cancerous cells than in the paired non-cancerous cells.

### Correlation between hPer2 protein expression and clinical-pathological features in colorectal cancer patients

In order to establish the clinical and pathological relevance of low expression of hPer2 protein in human colorectal cancer, we assessed the relationship between hPer2 expression and clinical-pathological parameters. The results are shown in table 2 table 3 and table 4. Decreased hPer2 expression were found in all the younger patients with age no more than 50 while only 53% patients in the older group had low hPer2 expression. However, no correlations were found between low hPer2 expression and sex or tumor site.

**Table 2:** Relationship between expression levels of hPer2 protein in colorectal carcinoma and clinical features

clinical-pathological features	Total cases n	Decreased hPer2 expression in tumor n(%)	P
Sex			0.357
male female	18 20	10(55.6) 14(70. 0)	
Age			0.017
≤50 > 50	8 30	8(100.0) 16(53.3)	
Tumor site			0.393
colon rectum	21 17	12(57.1) 12(70.6)	

**Table 3:** Relationship between expression levels of hPer2 protein in colorectal carcinoma and pathological features

clinical-pathological features	Decreased hPer2 expression in tumor n	Non-Decreased hPer2 expression in tumor n	P
Pathology Type			0.235
protrude infiltrating ulcer	7 3 14	8 1 5	
Histological Grade			0.017
I, II III	16 8	14 0	
Depth of Tumor Invasion			0.044
T_is_~T_2_ T_3_~T_4_	3 21	6 8	
Lymph Nodes Spread			0.043
negative positive	10 14	11 3	
TNM Stage			0.021
I II III+IV	2 8 14	6 5 3	
p53			0.167
(-) (+)	2 22	4 10	
C-erB-2			0.804
(-) (+)	13 11	7 7	

**Table 4:** Relationship between the reduced expression of hPer2 protein and Ki67 in colorectal cancer patients

low hPer2 expression in tumor	Ki67		
	
	n	$\bar{x}$ ± s	P
Negative	14	0.476 ± 0.262	0.046
Positive	24	0.638 ± 0.214	

Low hPer2 expression is also found to be correlated with higher histological grade (p < 0.017), deeper tumor invasion (P < 0.044), positive lymph nodes metastasis (P < 0.043) as well as more advanced TNM stage (P < 0.021). In order to further understand the potential role of hPer2 at molecular level, Ki67, a proliferation marker, was also evaluated. And we found that tumors with low hPer2 expression displayed higher Ki67 score than that without, consistent with the notion that losing hPer2 expression may promote cancer cell growth. However, there were no statistical differences between hPer2 expressions with expressions of p53 or C-erB-2.

### Determination of hPer2 mRNA level by real-time PCR

**Figure 3:**
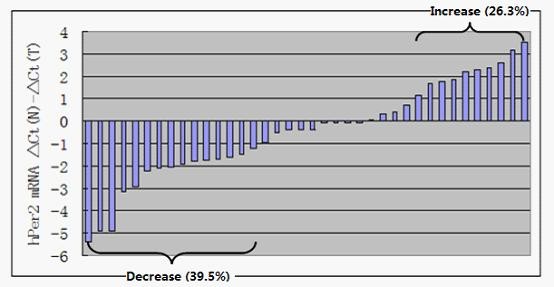
**Determination of hPer2 mRNA by real-time PCR**. ΔCt (N): Ct value of GAPDH was subtracted from Ct value of hPer2 of non-cancerous tissues. ΔCt (T): Ct value of GAPDH was subtracted from Ct value of hPer2 of CRC. Bar value(ΔCt (N)- ΔCt (T))represents the difference of hPer2 mRNA between non-cancerous tissue and paired CRC tissue. Each bar represented for one sample. Bar value = -1 indicates that hPer2 mRNA of CRC is 2^-1^-fold of that of paired non-cancerous tissue. Bar value = 1 indicates that hPer2 mRNA of CRC is 2^1^-fold of that of paired non-cancerous tissue. Bar value≤-1 indicates that the expression of hPer2 is decreased in tumors. Bar value≥ 1 indicates that the expression of hPer2 is increased in tumors.

We further analyzed hPer2 mRNA levels in these 38 paired cancerous and non-cancerous tissues by real-time PCR. The amount of hPer2 mRNA was normalized using the endogenous reference GAPDH. The normalized hPer2 mRNA expression (ΔCt) of tumor tissue was then compared with the ΔCt of the paired non-tumor tissues from the same colorectal cancer patient to determine their relative expression levels (ΔΔCt) (Figure 3). Then we analyzed hPer2 mRNA expressions in these 24 paired tissues which showed low expression of hPer2 protein in tumor. Our results showed that the mRNA levels of hPer2 in colorectal cancer were decreased compared with those in paired non-tumor tissues (P < 0.05). The relative expression levels (2^-ΔΔCt^) of hPer2 in colorectal cancer compared with non-cancerous components were 1: 1.219.

## Discussion

Studies in breast cancer and hepatic carcinoma have suggested that disturbed hPer2 gene expression is associated with human tumor progression [26,27]. Genetic studies then have provided direct evidence to show that mPer2 is a tumor suppressor in mice [10,28]. Whether this clock gene Per2 serves a similar role in human CRC is unclear. Aiming to investigate this question directly, in this study we first examined the protein expression of hPer2 in colorectal cancer patients. In order to make the cancerous and non-cancerous tissues be synchronized to the same circadian clock and be comparable, we analyzed and compared the expression status of the hPer2 proteins between cancerous and non-cancerous cells of the same patient sample. The results revealed that hPer2 protein expressed in both colorectal carcinoma tissues (31 cases of the total 38 cases) and surrounding non-cancerous tissues (37 cases of the total 38 cases). By further comparisons of the average level of hPer2 protein expression in the paired tissues, significantly decreased hPer2 expression in these cancerous tissues were found (24 cases of the total 38 cases). In mammals, the rhythmic expression of Period genes is essential for creating a beating clock [2,29]. Homologues of most of the genes involved in the fly circadian clock have been cloned in mammals, and the general core clock mechanism of interacting transcriptional feedback loops is similar between flies and mice [30]. Abundant evidences generated from mammalian circadian rhythms have indicated that disruption of mPer2 genes causes immediate behavioral arrhythmicity of mPer2 knock-out mouse [31-33]. Since the Per2 gene expression level decreased in the CRC tissues, our results may suggest that the circadian clock in most human CRC cases behaves arrhythmic, and differently from that in non-cancerous cells.

Immunohistochemical analyses also showed differential expression patterns of hPer2 protein in our samples. Most colorectal cells in non-cancerous tissues were homogeneously stained. In contrast, in the paired cancerous tissues, heterogeneous staining manifested commonly. The cancerous cells were positively or strongly stained in some areas while negatively or weakly stained in other areas of the same tissue. It suggests that several asynchronized circadian clocks may operate in different sub-populations within the same tumor simultaneously. Furthermore, in cancerous tissues, well-differentiated cells seem to have higher level of hPer2 compared to that in the poorly-differentiated cells. This phenomenon may suggest that inactivation of hPer2 not only correlates with colorectal tumor initiation but also plays a role in tumor progression.

We further analyzed hPer2 expression patterns with clinical-pathological features of these colorectal tumor tissues. We found that cancerous tissues with high grades of histopathological changes and advanced stages of TNM classification showed statistically weaker hPer2 protein expression, especially in those patients under 50. Since these clinical-pathological features correlate with relatively poor prognosis of colorectal cancer patients, low hPer2 expression may be utilized as one of the prognostic biomarkers for colorectal cancer and worth further investigation in a larger sample set. In our study, a relation of low expression of hPer2 in tumor tissues with strong Ki67 was also found. It suggests that deregulated expression of hPer2 may contribute to the highly proliferative property of colorectal cancer cells. Previous studies have shown that aberrant expression of circadian clock genes could have important consequences on the transactivation of downstream targets that control the cell cycle and on the ability of cells to undergo apoptosis, potentially promoting carcinogenesis [1,2]. C-erB-2 and p53 were shown to be partly controlled by hPer2 in the course of tumor progression [6,10]. However, in this study, we observed no statistically significant association between low levels of hPer2 expression and positive p53 or C-erB-2, which need to be further investigated in a larger sample set.

Recently, more direct evidences demonstrate the link between the loss of circadian genes function and cancer progression, but the underlying mechanisms were poorly understood. Chen ST. et al. reported that 95% of breast tumors (56 out of 59 specimens) displays no or deregulated level of Per1, Per2 or Per3 proteins in the breast tumor cells when compared the adjacent normal tissues [6]. Yeh et al. found that the expression level of Per1 was significantly decreased in endometrial carcinoma [34]. In these studies, the loss of clock gene expression was due to DNA methylation of the promoters rather than gene mutations of the clock genes. Later, Remco et al. identified the miR-192 ⁄ 194 cluster as a potent inhibitor of the entire Period gene family using a forward genetic screen, unveiling a new mechanism for the downregulation of the circadian clock genes at the post-transcriptional level [35]. To elucidate a possible mechanism to explain the hPer2 expression patterns in colorectal cancer, we further analyzed hPer2 mRNA levels in these 24 paired tissues which showed low expression of Per2 protein in tumor. Our results showed that the mRNA levels of Per2 were also decreased in cancerous tissues. It suggests that other factors rather than miR-192⁄194 cluster may be responsible for the hPer2 gene deregulation in CRC progression. Since it has been shown that CpG methylation can inactivate promoter function, leading to inhibition of hPer gene promoter function in breast and endometrial carcinoma, whether CpG methylation on the PER2 promoter contributes to human colorectal cancer development needs further investigation.

Both genetic and molecular studies have shown that hPer2 is a tumor suppressor that controls cell proliferation and promotes apoptosis. Our data suggest that aberrant expression of the hPer2 gene may be associated with the development of carcinomas in the colorectal tissues and may provide a molecular basis for the clinical application of chronotherapy in CRC treatment. However, the mechanisms leading to the reduced expression of hPer2 and the manner in which hPer2 suppresses tumorigenesis remains unclear. Further studies are still necessary to elucidate the role of hPer2 in CRC.

## Conclusions

Our study showed hPer2 expression decreased in human CRC tissues, and well-differentiated cancer cells are more likely to maintain hPer2 expression than poorly-differentiated ones. Furthermore, associations of decreased hPer2 levels with patients' age, histological grade, TNM stage and expression of nucleus proliferation related antigen: Ki67 were also detected. These results suggest a role for hPer2 in normal colorectal cell function and the potential deregulation of hPer2 expression in the development, invasion, and metastasis of CRC.

## Competing interests

The authors declare that they have no competing interests.

## Authors' contributions

WYP collected the cases and clinical information, assisted in performing the study experiments, performed the statistical analysis and drafted the manuscript. LC carried out the immunoassays, analyzed pathological slides, and performed quantitative RT-PCR studies. CZY participated in the study's design and coordination. HLC conceived the study, pooled resources to perform the study experiments, performed study experiments, pathological slides analysis, preparation of manuscript, and editorial review, final editing and proofing prior to submission as corresponding author. All authors read and approved the final manuscript.
